# Three‐dimensional environment and vascularization induce osteogenic maturation of human adipose‐derived stem cells comparable to that of bone‐derived progenitors

**DOI:** 10.1002/sctm.19-0207

**Published:** 2020-07-08

**Authors:** Amel Ibrahim, Naiara Rodriguez‐Florez, Oliver F. W. Gardner, Eleonora Zucchelli, Sophie E. P. New, Alessandro Borghi, David Dunaway, Neil W. Bulstrode, Patrizia Ferretti

**Affiliations:** ^1^ Stem Cells and Regenerative Medicine Section UCL Great Ormond Street Institute of Child Health London UK; ^2^ Department of Plastic Surgery Great Ormond Street Hospital for Children NHS Foundation Trust London UK; ^3^ TECNUN Escuela de Ingenieros Universidad de Navarra San Sebastian Spain

**Keywords:** 3D environment, bone, differentiation, human adipose‐derived stem cells, mesenchymal stem cells, osteogenesis, progenitor cells, tissue engineering, vascularization

## Abstract

While human adipose‐derived stem cells (hADSCs) are known to possess osteogenic differentiation potential, the bone tissues formed are generally considered rudimentary and immature compared with those made by bone‐derived precursor cells such as human bone marrow‐derived mesenchymal stem cells (hBMSCs) and less commonly studied human calvarium osteoprogenitor cells (hOPs). Traditional differentiation protocols have tended to focus on osteoinduction of hADSCs through the addition of osteogenic differentiation media or use of stimulatory bioactive scaffolds which have not resulted in mature bone formation. Here, we tested the hypothesis that by reproducing the physical as well as biochemical bone microenvironment through the use of three‐dimensional (3D) culture and vascularization we could enhance osteogenic maturation in hADSCs. In addition to biomolecular characterization, we performed structural analysis through extracellular collagen alignment and mineral density in our bone tissue engineered samples to evaluate osteogenic maturation. We further compared bone formed by hADSCs, hBMSCs, and hOPs against mature human pediatric calvarial bone, yet not extensively investigated. Although bone generated by all three cell types was still less mature than native pediatric bone, a fibrin‐based 3D microenvironment together with vascularization boosted osteogenic maturation of hADSC making it similar to that of bone‐derived osteoprogenitors. This demonstrates the important role of vascularization and 3D culture in driving osteogenic maturation of cells easily available but constitutively less committed to this lineage and suggests a crucial avenue for recreating the bone microenvironment for tissue engineering of mature craniofacial bone tissues from pediatric hADSCs, as well as hBMSCs and hOPs.

## Graphical abstract

Human adipose‐derived stem cells (hADSCs) are a more accessible source of cells for bone tissue engineering but limited by immature osteogenic differentiation capacity. This study demonstrates that three‐dimensional microenvironment and early vascularization promote osteogenic maturation achieved by hADSC comparable to calvarial bone‐derived precursor cells such as human bone marrow‐derived mesenchymal stem cells (hBMSCs) and human osteoprogenitor cells (hOPs).

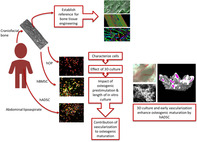

**Significance statement**

Tissue‐engineered bone can provide a lifelong solution for reconstructing deformities and defects in the pediatric facial skeleton; thus, bypassing the risk of infection and invasive surgery associated with current treatments. Fat‐derived stem cells are an abundant and easily isolated source for bone tissue engineering. So far, they have been limited by the immaturity of the bone formed. This study demonstrated that altering the physical environment and introducing a blood supply can enhance the maturity of the bone these cells form. This provides the foundation for engineering more advanced bone to provide personalized replacement tissues.

## 1 INTRODUCTION

Reconstruction of pediatric craniofacial bone defects usually requires invasive transfer of bone (often calvarial grafts). Alternative treatments such as foreign‐body implants neither resorb nor produce a secondary donor‐site defect like bone grafts. They are however associated with increased risk of infection, extrusion, and may not grow or integrate with the skeleton. Tissue engineering holds the promise of generating autologous replacement tissues without these limitations. Nonetheless, there are still a number of critical barriers to engineering clinically appropriate bone.

Several tissues contain mesenchymal stem cells (MSCs) which are capable of self‐renewal and can undergo skeletogenic differentiation.^1^, ^2^, ^3^ Human bone marrow‐derived mesenchymal stem cells (hBMSCs) are considered MSCs with high osteogenic potential.^4^ They also showed promise in repairing subacute critical sized long bone defects in a large animal model and successfully regenerated a maxillary oral defect in a clinical study.^5^, ^6^ Human osteoprogenitor cells (hOPs) are another promising source of MSCs that can be obtained from a variety of bone tissues and give rise to osteoblast‐like cells.^7^, ^8^ They may also possess a similar multipotent and proangiogenic profile to hBMSCs in children.^9^, ^10^ Human adipose‐derived stem cells (hADSCs) are another important MSC source for bone engineering due to their multilineage differentiation potential,^11^, ^12^ immune‐modulatory properties, and ability to promote vascularization.^13^, ^14^ Furthermore, harvesting of hADSCs is considerably less invasive than hBMSCs or hOPs, which require a bone biopsy. hADSCs have been successfully used to repair critical bone defects in rats.^15^, ^16^ To date however, hADSCs have shown considerably less osteogenic ability compared with hBMSCs in vivo.^17^ Given their many desirable characteristics, it would be important to maximize hADSC osteogenic potential for bone bioengineering.

A strategy to increase osteogenic potential is the use of biomaterials and/or matrix proteins to recreate a stem cell niche that could enhance cell adhesion, proliferation, and differentiation.^18^, ^19^ Fibrin is an attractive biomaterial for this purpose; it has been repeatedly shown to promote survival, proliferation, and osteogenic differentiation as well as support vascularization.^20^, ^21^ It can also be loaded with osteoinductive agents to stimulate osteogenesis as evidenced when implanted in rat tibial bone defects.^22^ Furthermore, it has been used to deliver osteogenically differentiated hBMSC with bone grafts to unite nonhealing fractures in patients.^23^

The aim of this study was to compare the potential of pediatric hADSCs to hBMSCs and hOPs for generating mature bone using pediatric calvarial bone as a reference, as well as devising strategies for increasing osteogenic maturation particularly in hADSCs. Hence, first, we further characterized pediatric calvarial bone and second studied the impact of differentiating MSCs in three‐dimensional (3D) fibrin scaffolds in vitro and after grafting them onto the chick chorioallantoic membrane (CAM). Over the time frame used in this study, we showed that both 3D culture and a short period of vascularization enhanced hADSCs maturation. Together with novel information on the organization of the extracellular matrix in native bone, this study shows that investigating 3D culture and vascularization of hADSCs could open new opportunities for bone tissue engineering.

## 2 METHODS

All chemicals and reagents were purchased from Sigma‐Aldrich (Haverhill, UK) unless otherwise stated. All cells were cultured in humidified incubators at 37°C with 5% CO_2_.

### 2.1 Human tissues

Tissues were collected from consented patients undergoing elective reconstructive surgery (at Great Ormond Street Hospital for Children) under ethical approval from the Camden and Islington Community Local Research Ethics Committee (London, UK).

### 2.2 Culture of cells from human adipose and bone tissues

Cell types used and their sources are listed in Table S1. All cells were grown in expansion medium consisting of high glucose Dulbecco's modified Eagle's medium containing GlutaMAX, 10% embryonic stem cell‐qualified fetal bovine serum, and 1% penicillin/streptomycin (Life Technologies, Warrington, UK). Induction of differentiation along adipogenic, chondrogenic, and osteogenic lineages was carried in confluent cultures as previously described.^12^, ^24^ Expansion media was either used alone (control) or as a base for adipogenic (1 ng/mL insulin, 500 μM 3‐isobutyl‐1‐methylxanthine, 1 μM dexamethasone, and 1 μM rosiglitazone), chondrogenic (0.1 μM dexamethasone, 10 ng/mL transforming growth factor β1, insulin transferrin selenium, and 50 μg/mL ascorbate‐2‐phosphate), or osteogenic (0.1 μM dexamethasone, 100 mM β‐glycerophosphate, and 100 μg/mL ascorbate‐2‐phosphate) media. Live cells were imaged using an inverted microscope Olympus IX71 with a Hamamatsu ORCA‐ER digital camera (Hamamatsu Corp) using HCImage processing software.

#### 2.2.1 *Human adipose tissue‐derived stem cells*

Surplus lipoaspirates from the abdominal subcutaneous tissues were taken from consented patients undergoing lipofilling procedures. hADSCs (n = 12) were isolated either from explants or by enzymatic digestion as previously described.^12^

#### 2.2.2 *hBMSCs and hOPs*

Bone samples routinely discarded during sagittal spring insertion surgery were collected from children with nonsyndromic sagittal craniosynostosis to isolate hBMSCs (n = 7) and hOPs (n = 11). Bone samples washed in phosphate‐buffered saline (PBS; PAA Laboratories Ocoid, Basingstoke, UK) were cut into pieces, the bone marrow cavities flushed with sterile PBS until white, and the cell suspension centrifuged at 1000 rpm for 5 minutes. The remaining hBMSC pellet was resuspended and cultured in expansion medium. The flushed bone fragments were cut (1 × 1 mm in size); 2 to 3 pieces/well were plated as explants then covered with expansion media to isolate hOPs.

### 2.3 3D cell culture on fibrin

Cells were suspended in sterile bovine thrombin solution (100 iU/mL) at 10^5^ cells/gel and combined with fibrinogen 10 mg/mL to obtain a 100 μL gel. Cellularized scaffolds were allowed to polymerize for 30 minutes in an incubator before adding expansion medium.

### 2.4 CAM grafting

CAM‐grafting was carried out under the Animals Scientific Procedures Act 1986 using fertilized brown Leghorn eggs (Needle Farm, UK) as previously described.^25^ Pediatric hADSCs encapsulated in 2 mm fibrin scaffolds were cultured in control or osteogenic differentiation media for 2, 3, or 4 weeks prior to CAM grafting for 7 days and analyzed in parallel with scaffolds maintained in culture for the total length of the experiment (3, 4, and 5 weeks). Cells from three different patient lines were used (n ≥ 3 per line for each condition). After imaging the graft in ovo, CAM‐grafted scaffolds were harvested, washed in PBS, and reimaged before fixation.

### 2.5 Viability and proliferation assay

Freshly plated cells (n = 8 per cell line; n = 3 cell lines per group) were cultured for 4 hours before incubating with 20% Resazurin for 4 hours then analyzing media at 569 and 595 nm with a plate reader. Assay was repeated at 24, 48, 72 hours and 7 days.

### 2.6 Gene expression analysis by reverse transcription polymerase chain reaction

RNA was isolated using TRizol (Life Technologies) as per manufacturer's protocol. All cDNA was made from 1ug of RNA through retrotranscription with Moloney murine leukemia virus reverse transcriptase (M‐MLV; Promega, Madison, Wisconsin). Reverse transcription polymerase chain reaction was performed using primers targeting genes of interest (Table S2). Real time semi‐quantitative polymerase chain reaction (RT‐qPCR) was performed using the ABI Prism 7500 sequence detection system (Applied Biosystems, California) with the Quantitect SYBR Green PCR kit (Qiagen, Hilden, Germany). All samples were run in technical and biological triplicates (n = 3 individual lines per cell type). Data were analyzed using the ΔΔCT method using human bone as a control.

### 2.7 Flow cytometry

Undifferentiated hADSCs (n = 3), hOPs (n = 5), and hBMSCs (n = 5) at passages 3 to 8 were incubated with blocking solution (2.5% fetal bovine serum in PBS) for 30 minutes, followed by 15 minutes at 4°C with antibodies (Table S3), then washed in PBS. Negative controls were incubated with the isotype control. Flow cytometry was performed using FACSCalibur (BD Biosciences, California) with Cell Quest Pro software. Data were analyzed using FCS Express 6.0 (De Novo Software, Glendale, California).

### 2.8 Histochemistry, immunofluorescence, and quantification of osteogenic differentiation

Cellularized scaffolds were fixed in 4% paraformaldehyde, cryoembedded, and 10 to 30 μm sections cut. Bone samples were decalcified in 15% EDTA tetrasodium salt dihydrate for up to 12 days at 4°C, paraffin embedded, and 4 μm sections cut. Sections were deparaffinized using Histo‐Clear (National Diagnostics) and rehydrated prior to analysis.

To assess adipogenic and chondrogenic differentiation, cells were stained with oil Red O and alcian blue, respectively. To detect osteogenic differentiation tissues and cells were stained using alizarin red or Von Kossa and MacNeal's tetrachrome costaining. Images were taken using the Axiovert 135 (Zeiss, Welwyn Garden City, UK) with a ProgRes C14 digital camera using and processed using Openlab software (PerkinElmer Life, Wokingham, UK).

#### 2.8.1 *Fluorescence and second harmonic generation scanning*

Immunofluorescent staining and nuclear counterstaining with Hoechst dye were performed as previously described^25^; the antibodies and dilution used are shown in (Table S4). Fluorescently labeled cells were imaged using an inverted microscope Olympus IX71 (Zeiss) equipped with a Hamamatsu ORCA‐ER digital camera (Hamamatsu Corp) using HCImage processing software. Confocal microscopy imaging was performed using the Zeiss LSM 710 upright confocal and Zen software (Zeiss, Welwyn Garden City, UK). The ParticleAnalyzer plugin on ImageJ^26^ was used to compare nuclear size and circularity.

The upright Zeiss LSM 880 multiphoton was used to image whole mount samples with accompanying Zen software (Zeiss). The multiphoton was used to perform second harmonic generation scanning (SHG) to visualize collagen fibers. Imaging of collagen either following immunoflourescent staining or SHG was analyzed using ImageJ software. All images were postprocessed using the despeckle, background subtraction, and Fourier transformation filters. Quantitative and qualitative analysis of collagen fiber orientation was performed on SHG images using OrientationJ plugin. Fifty regions of interest (ROIs) selected in sequence per bone section (a total of n = 3 per patient) were analyzed using a uniform weighting which provided the coherency; a measure of isotropy and orientation of the collagen fibers in that area. Coherency ranges from 0 (anisotropic) to 1 (isotropic).

#### 2.8.2 *Micro‐computed tomography*

High‐resolution micro‐computed tomography (μCT) scanning was performed using the Skyscan1172 μCT scanner (Bruker, MicroCT, Kontich, Belgium) with an aluminum filter for calvarial bones. The parameters used were an x‐ray tube voltage of 49 kV and a current of 200 μA, scanning angular rotation of 0°, and angular increment of 0.4°. Median filtering and flat‐field correction were applied to all scans to reduce ring artifacts. Scans were reconstructed with NRecon software (version 1.6.6.0, Skyscan N.V., Kontich Belgium) using the Feldkamp algorithm. All scans were optimized using beam‐hardening correction, alignment optimization, and ring artifact correction during reconstruction. The resultant image resolution was 3.88 μm.

Postprocessing of two‐dimensional (2D) stacks of the reconstructed images was performed using ImageJ. Stacks were converted to 8‐bit gray scale then underwent the despeckle filter, background subtraction using a sliding ball algorithm and the 3D Gaussian blur filter. The WEKA trainable segmentation plugin for ImageJ was used to segment the samples.^27^ For each stack, an area enclosing the whole scaffold(s) was cropped using a square ROI and pixels were classified as either scaffold, blood vessel, mineral, or background as identified by the user to train the classifier. The 3D viewer was used to build 3D models to visualize mineralization and vascularization of the scaffolds.

### 2.9 Statistical analysis

Unless otherwise stated, all experiments were conducted using experimental triplicates using at least three individual cell lines. All data are presented as the mean ± SEM. Statistical significance was evaluated using analysis of variance (ANOVA) followed by a Tukey's post hoc test using GraphPad Prism 7.0 (GraphPad, La Jolla, California). A *P* ≤ .05 was considered statistically significant.

## 3 RESULTS

### 3.1 Structural and biomolecular characterization of pediatric calvarial bone

Histological composition, mineralization, and protein expression of calvarial bone from 11 patients were studied to define criteria for evaluating tissue engineered craniofacial bone.

**FIGURE 1:**
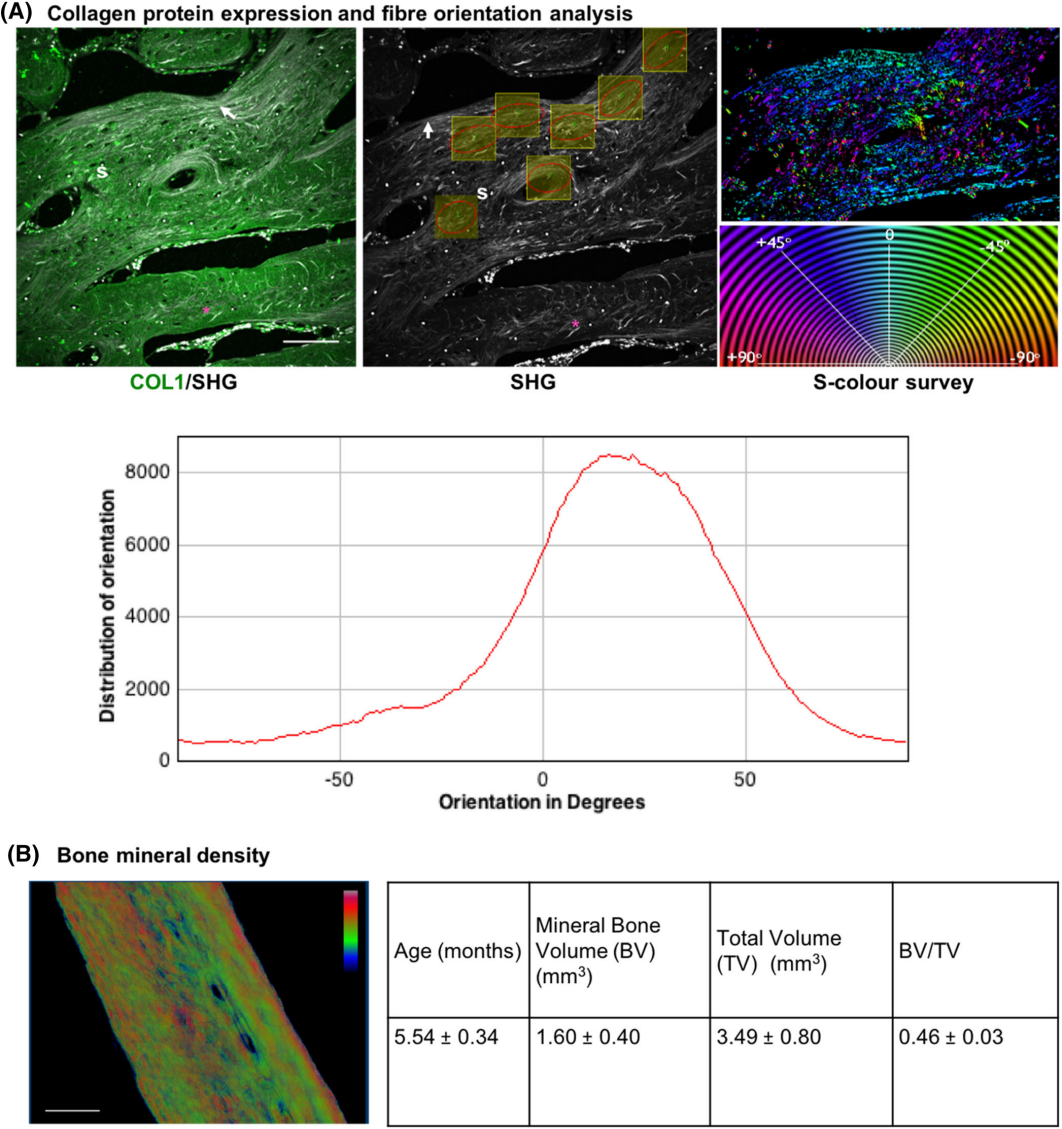
Collagen expression, fiber orientation, and bone mineral density analysis of pediatric human calvarial bone. A, Collagen‐1 (COL1) detection by immunofluorescent staining and SHG imaging of collagen on bony spicules, osteoid (arrows), and osteoblast containing matrix (*). Analysis of SHG images shows collagen networks in gray scale, coherency across multiple regions of interest, and S‐color map with accompanying fiber orientation color wheel of local fiber orientation in degrees (−90° to 90°). The bottom panel shows the histogram of distribution of fiber orientations; note osteoid fibers predominantly aligned between 0° and 90°, while collagen orientation in the spicules is more heterogeneous. B, μCT analysis of mineralization density; 3D reconstructions of the μCT slices were used to visualize the bone extracellular matrix structure. The μCT stacks of bone were thresholded on ImageJ to isolate the mineralized tissue and revealing the bone structure and gray density values. The thermal color filter visually represents the gradient of mineral density with red correlating with increased mineral density and blue coinciding with reduced mineral density. The numerical values show quantification of bone volume (mineralized matrix, BV), total volume (mineral and organic components, TV) and the ratio between them, the bone fraction volume (BV/TV), assessed using BoneJ (n = 11). Scale bars (A) = 100 μm, scale bars (B) = 0.5 mm. μCT, micro‐computed tomography; BV, bone volume; l, lacuna; s, spicule; SHG, second harmonic generation; TV, total volume

Collagen‐1 (COL1) immunostaining revealed widespread collagen deposition in bone islands (spicules) and in the immature bone (osteoid) layer, the extracellular matrix freshly deposited by osteoblasts (Figure 1A). This was consistent with SHG scanning; calvarial samples contained an abundant extracellular collagen meshwork with fibrils tightly packed and aligned in parallel to the long axis of the bone at the osteoid matrix; fibril density appeared looser with less polarized orientation in the spicules. In the ROI over the osteoid region, coherency was higher (0.37 ± 0.018) than in the spicules (0.25 ± 0.018) confirming a difference in collagen arrangement (*P* < .0001). The histogram of orientation throughout the sample suggests that orientation is overall skewed positively (0‐90°).

Calvarial bone (n = 11) density was analyzed by μCT (Figure 1B). A color thermal LUT was applied to visually represent the gradient of mineralization density in calcified samples. Higher mineralization density was observed at the bone cortical surface (red‐green) than at its core (green‐blue), consistent with the presence of more densely packed collagen fibers in the cortex. Quantification of bone volume (mineralized matrix) and total volume (mineral and organic components) was carried out and bone fraction volume (BV/TV), a measure of mineralized bone density, calculated using BoneJ.^28^ The average BV/TV was 0.46 in the data set of calvarial bone from children aged 3.7 to 7.4 months old in line with previous studies by our group.^29^

### 3.2 Characterization of isolated hADSCs, hOPs, and hBMSCs

**FIGURE 2:**
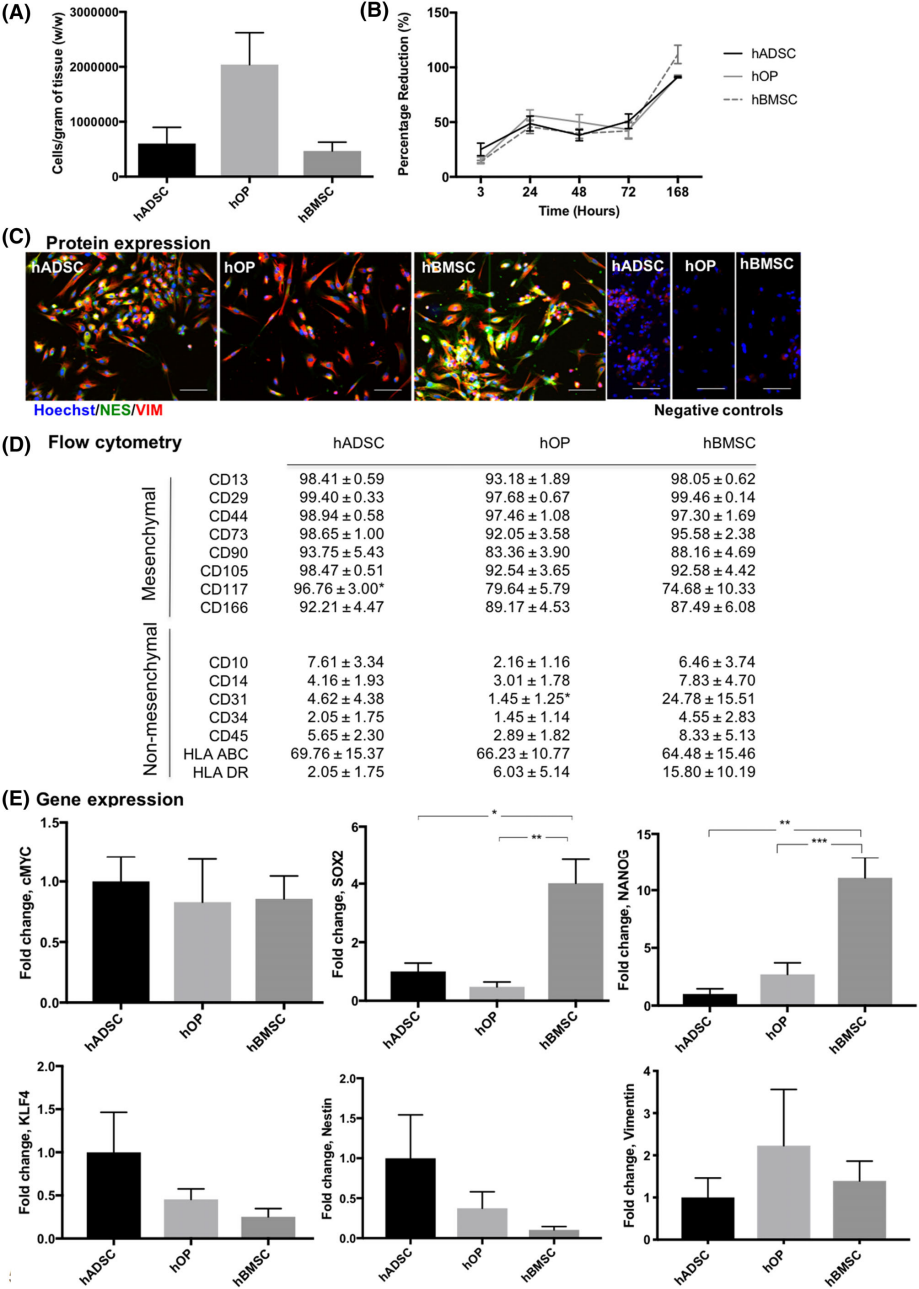
Comparison of different pediatric osteoprogenitor cell types using flow cytometry, gene expression, protein expression, and proliferative capacity. A, Cells yielded per gram (wet weight) of tissue assessed by counting the number of retrieved cells: hADSCs (n = 3, 603 970 ± 293 610), hOPs (n = 5, 2 042 000 ± 580 825), and hBMSCs (n = 4, 468 114 ± 160 122). B, Proliferation of osteoprogenitor cell types using Alamar blue assay; note a significant difference in proliferation rate of hBMSC between 72 and 168 hours compared with hADSC (*P* = .0268) and hOP (*P* = .0317). C, Immunoflourescent staining for NES and VIM protein expression by hADSC (n = 2), hOP (n = 3), and hBMSC (n = 3) cells on monolayer culture in expansion media. Images representative of staining from different areas on coverslip and cell lines from hADSC (n = 2), hOP (n = 3), and hBMSC (n = 3). D, Panel of mesenchymal and nonmesenchymal markers used to analyze cell lines from different patients to compare hADSC (n = 3), hOP (n = 5), and hBMSC (n = 5). Comparison of hADSC vs hOP, hADSC vs hBMSC, or hOP vs hBMSC. E, Relative quantification PCR result of expression of pluripotency markers for hADSC (n = 3), hOP (n = 5), and hBMSC (n = 5) presented as fold change relative to hADSC. Nuclei counterstained with Hoechst (blue), negative controls (no primary antibody) for Alexa Fluor 488 nm anti‐rabbit (green) and Alexa Fluor 594 nm anti mouse (red). Scale bars = 100 μm. All data are expressed as mean ± SEM, statistical significance: *P < .05, ****P < .0001. hADSCs, human adipose‐derived stem cells; hBMSCs, human bone marrow‐derived mesenchymal stem cells; hOPs, human osteoprogenitor cells; NES, nestin; PCR, polymerase chain reaction; VIM, vimentin

There was no significant difference in cell yield per wet weight of explanted fat, bone, and bone marrow (Figure 2A). Proliferation of hADSCs, hOPs, and hBMSCs studied with Resazurin assay showed no significant difference up to 3 days (Figure 2B). In contrast, at 7 days hBMSCs had increased significantly more than hADSCs (*P* = .0268) and hOPs (*P* = .0317).

Morphology and behavior of hADSC, hBMSC, and hOP (Table S1; Figure S1) were compared. After two passages, hADSC cultures were mainly fibroblastic with a few flatter and polyhedral shaped cells also present, with no condensation observed at confluence. Also, hBMSCs predominantly comprised spindle‐shaped cells, which, like hOPs, tended to cluster and condense at confluence. Staining for nestin and vimentin, proteins usually associated with MSCs, showed that they were coexpressed in most hADSCs and hBMSCs, while hOPs expressed predominantly vimentin (Figure 2C).

Expression of mesenchymal and nonmesenchymal surface markers in hADSCs, hOPs, and hBMSCs was compared by flow cytometry (Figure 2D; Table S3).

All three progenitor cell types expressed typical mesenchymal markers (CD13, CD29, CD44, and CD73, CD90, CD105, CD166) with percentages all ≥83.36% ± 3.90%. The percentage of cells positive for CD117 (c‐kit), a marker expressed by a range of stem/progenitor cells, was significantly higher in hADSCs than in the other cell types (*P* = .0488), but above 74% average in all cases. Expression of nonmesenchymal markers (CD10, CD14, CD31, CD34, and CD45) was low in all groups, with CD31 significantly lower in hOPs than hBMSCs (*P* = .0119). Immunophenotype profile was compared using HLA‐ABC and HLA‐DR surface markers. While only a small percentage of HLA‐DR‐positive cells was present in all lines, the percentage HLA‐ABC‐expressing cells was around 60% in all.

Analysis by RT‐qPCR showed expression of cMYC, SOX2, KLF4, and NANOG in all cell types (Figure 2E). SOX2 and NANOG expression was significantly lower in hADSCs and hOPs than in hBMSCs, whereas cMYC and KLF4 did not significantly differ. All cell types expressed vimentin at similar levels and while nestin transcript was lower in hBMSCs than in the other cell type, this difference was not statistically significant (Figure 2E).

Trilineage differentiation capacity of all cell types was confirmed by culturing cells in control, adipogenic, chondrogenic, or osteogenic media for 3 weeks and staining with Oil‐red (fat droplets), alcian blue (chondroid matrix), and alizarin red (bone, calcium deposition). All lines could differentiate along these lineages, but alcian blue staining appeared more intense in hOPs and hBMSCs, while more lipid droplets were observed in adipogenically differentiated hADSCs (Figure S2). Mineralization was observed in all osteogenically differentiated cells.

### 3.3 Assessment of osteogenic potential of hADSCs, hBMSCs, and hOPs in monolayer cultures

**FIGURE 3:**
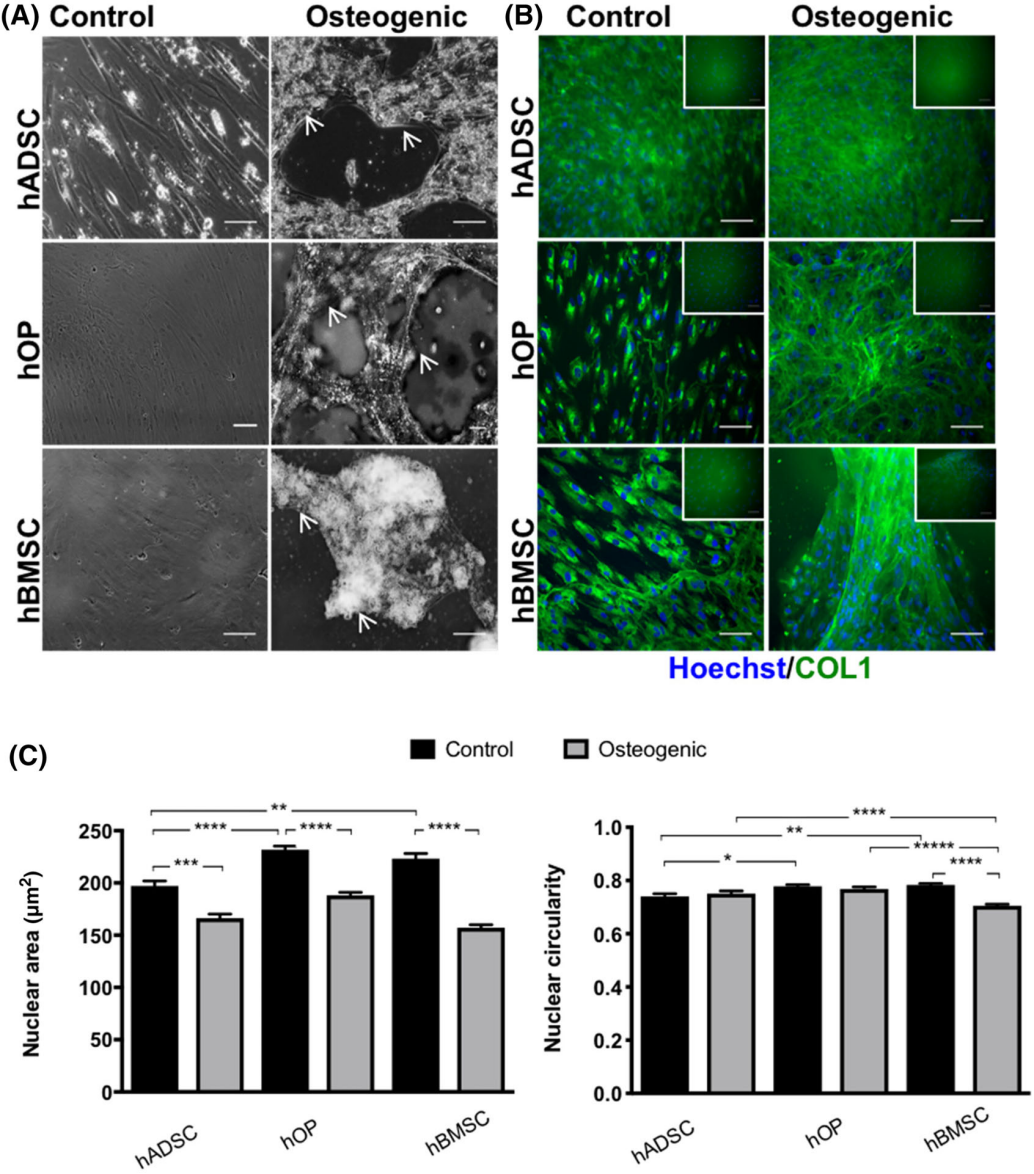
Comparison of osteogenic differentiation capacity of pediatric hADSCs adipose and bone derived osteoprogenitor cell types on monolayer culture using extracellular matrix analysis and nuclear morphometry. A, Phase contrast imaging illustrating cell aggregation and mineral nodule formation (white arrows) in differentiated hADSCs, hOPs, and hBMSCs on monolayer culture. B, Immunocytochemistry of Collagen‐1 (COL1, green) expression in control and differentiated cells. Insets show negative controls where the primary antibody was omitted. C, Nuclear morphometry comparing area and circularity of control (blacks bars) and differentiated (gray bars) cells assessed by segmenting Hoechst‐dye stained nuclei and analyzing them for area and circularity in ImageJ using the ParticleAnalyzer plugin. For nuclear circularity, roundness is measured from 0 to 1 (1 = perfect circle). Nuclei counterstained with Hoechst (blue, negative controls for Alexa Fluor 488 nm anti‐rabbit). Scale bars = 100 μm. Data expressed as mean ± SEM, statistical significance: *P < .05; **P < .01; ***P < .001, ****P < .0001. hADSCs, human adipose‐derived stem cells; hBMSCs, human bone marrow‐derived mesenchymal stem cells; hOPs, human osteoprogenitor cells

Osteogenic differentiation by the three cell types in monolayers was compared in greater depth after 3 weeks culture. Undifferentiated cells maintained a fibroblastic morphology and displayed little or no mineral or COL1 extracellular deposition. In contrast, differentiated cells aggregated to form multilayered structures with mineralized nodules and deposited COL1 extracellularly (Figure 3A,B).

Analysis of >150 cells per condition showed that nuclear size (area) of undifferentiated hADSCs was significantly smaller than that of control hOPs (*P* < .0001) and hBMSCs (*P* = .0011) (Figure 3C). Upon osteogenic differentiation, the nuclear size was significantly reduced in all cell types compared with their control counterparts (hADSC, *P* = .0002; hOPs, *P* < .0001; hBMSCs, *P* < .0001) but was greater in hOPs compared with hADSCs (*P* = .035) and hBMSCs (*P* < .0001). Nuclei of undifferentiated hADSCs exhibited a slightly lower circularity compared with hOPs (*P* = .0439) and hBMSCs (*P* = .006). Circularity was not affected by differentiation in hADSCs and hOPs but was reduced in differentiated hBMSCs compared to their undifferentiated controls and the other differentiated cell types (*P* < .0001).

### 3.4 Comparison of extracellular matrix formation and osteogenic maturation of hADSCs, hBMSCs, and hOPs in 3D culture

To assess whether a 3D environment could enhance osteogenic differentiation as compared to a 2D culture, cells were encapsulated in fibrin scaffolds and cultured in either control or osteogenic media for 3 weeks.

**FIGURE 4:**
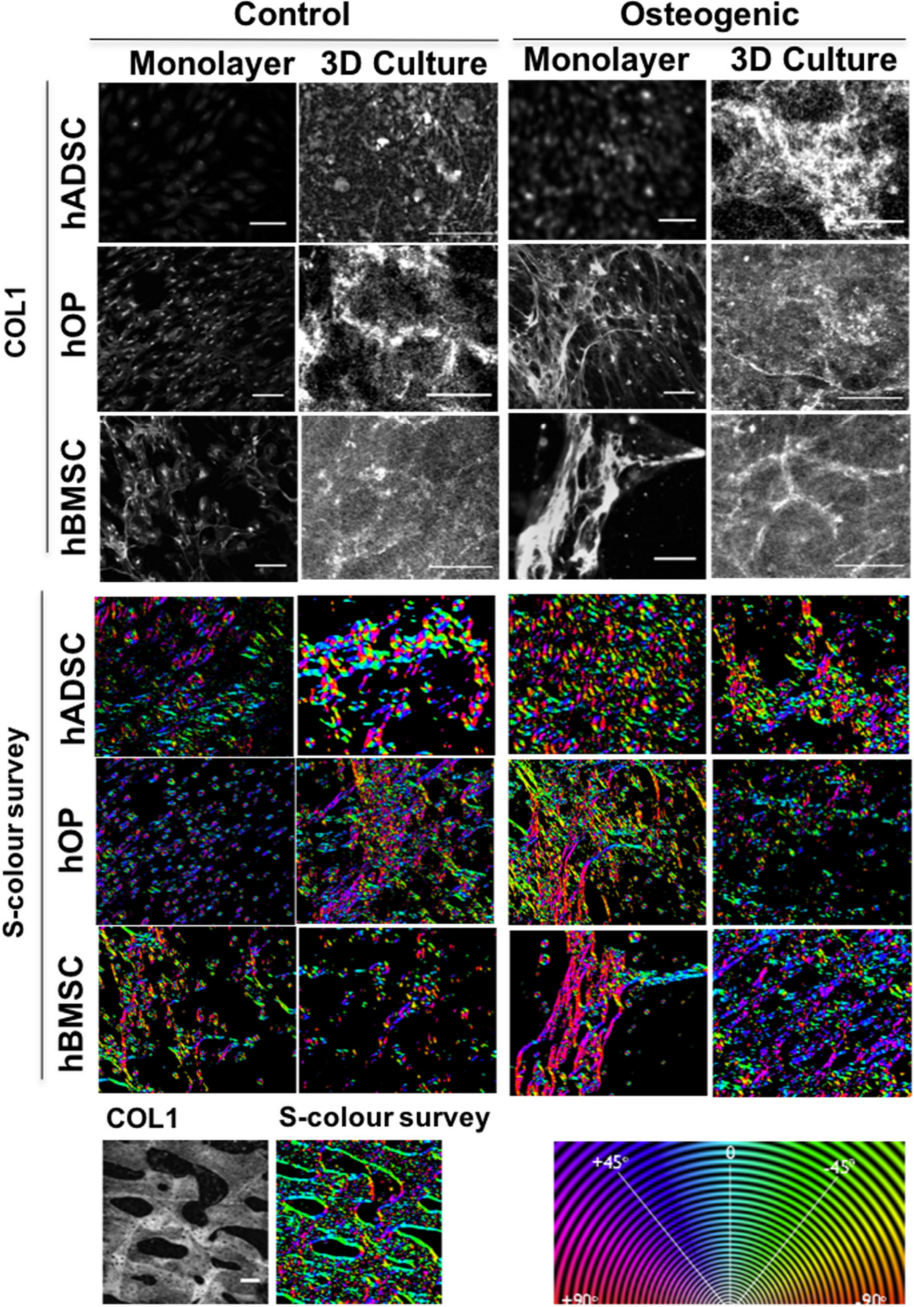
Comparison of collagen protein and gene expression and pattern of extracellular fibers by osteoprogenitor cells in monolayer and 3D culture. Immunoflourescent staining for Collagen 1 protein (gray) in hADSCs, hOPs and hBMSCs cultured in control or osteogenic medium for 3 weeks either in monolayer or fibrin gels (3D culture). Orientation and coherency of fibers assessed using OrientationJ: S‐color‐surveys demonstrate local collagen fiber orientations as represented by the color wheel. Collagen fiber orientation in bone is used as positive control. hADSCs, human adipose‐derived stem cells; hBMSCs, human bone marrow‐derived mesenchymal stem cells; hOPs, human osteoprogenitor cells

COL1 immunostaining was carried out in monolayer and 3D cultures (Figure 4). COL1 was evident in all osteogenically differentiated samples. COL1 was detected also in undifferentiated 3D samples, particularly hOPs and hBMSCs. Collagen networks were easily seen in calvarial bone, with S‐color‐surveys and histograms showing a relatively high amount of isotropy evidenced by the presence of fiber bundles and a single histogram peak (Figure S3). Extracellular and fibrillar distribution of collagen with greater isotropy after osteogenic differentiation was also seen in monolayer and 3D samples from all cell types, but with hBMSC fibrillar organization more closely resembling bone. To further quantify directionality of collagen fibers in 3D cultures, the coherency coefficient was assessed (Figure S4). Control and differentiated hADSCs and hOPs as well as undifferentiated hBMSCs had significantly lower coherency values than bone (*P* < .0001) suggesting lower isotropy. Osteogenic differentiation increased the coherency coefficient in hBMSCs (*P* < .0001) but not in hADSCs and hOPs when compared to controls.

Collagen 1 expression was used to compare three osteoprogenitor cell types in monolayer and 3D cultures (Figure S5). At baseline monolayer culture in control medium, Collagen 1 expression was significantly higher in hOPs compared with hADSCs (*P* = .0287). When the cells were cultured in 3D in control medium, there was no significant difference in Collagen 1 expression. Upon osteogenic differentiation in monolayer culture, Collagen 1 expression was significantly higher in hBMSCs than in hADSCs (*P* = .0051) and hOPs (*P* = .0079). In contrast, following osteogenic differentiation in 3D culture, no significant difference in Collagen 1 expression was observed between the three cell types.

### 3.5 Analysis of the effect of vascularization on osteogenic maturation of osteoprogenitor cells

**FIGURE 5:**
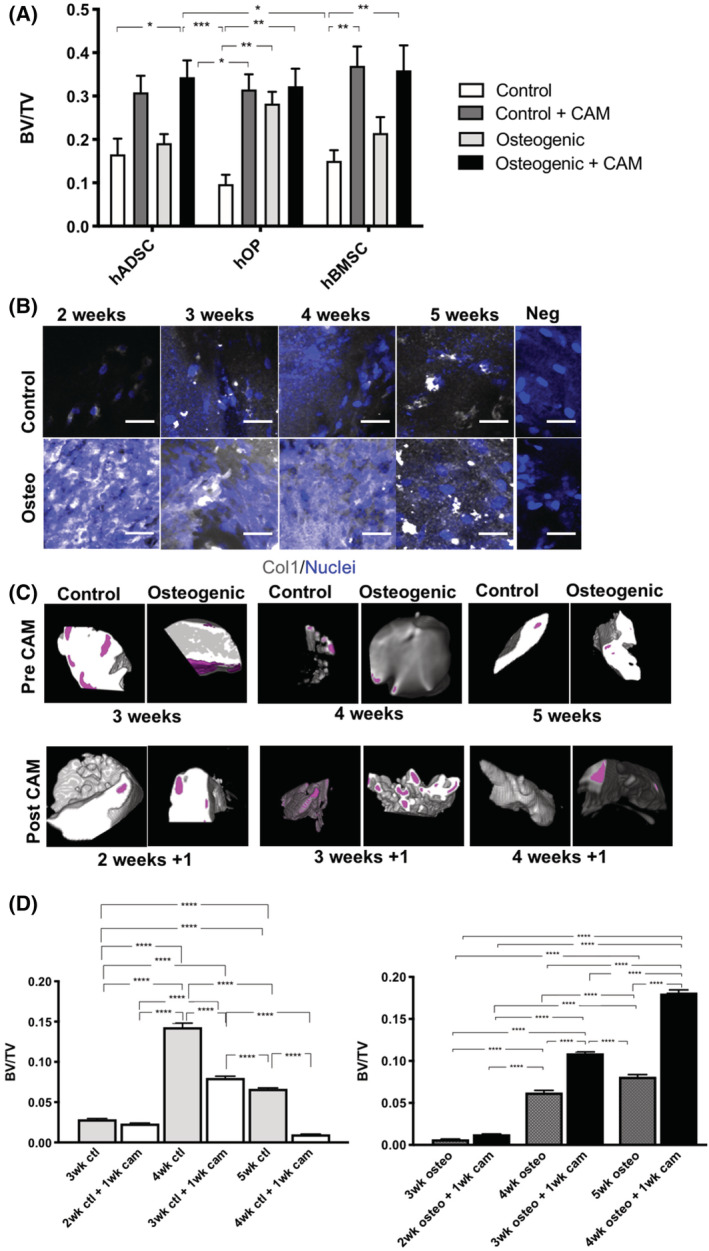
Mineralization and extracellular matrix deposition in hADSC, hOP, and hBMSC fibrin scaffolds in vitro and following CAM‐grafting. A, Mineralization and osteoid formation in cellularized hADSC, hOP, and hBMSC fibrin scaffolds maintained for 3 weeks in control or osteogenic medium and then either harvested for analysis or CAM‐grafted for 7 days; quantification is by MacNeal's tetrachrome stain intensity to determine the bone volume fraction (BV/TV). B, Immunoflourescent staining for Col1 in hADSC fibrin scaffolds cultured for different lengths of time showing increased staining under osteogenic differentiation conditions with greater extracellular matrix deposition in fibrillar network pattern with time peaking at 4 weeks in vitro. C, 3D models of surface mineral nodule formation (magenta) on hADSC fibrin scaffolds from the indicated differentiation time points before (pre) and after (post) CAM grafting for 7 days. D, Bone volume fraction (BV/TV) of μCT scans assessed in hADSC fibrin scaffolds cultured in vitro for 3, 4, or 5 weeks, or CAM‐grafted for 7 days following 2‐, 3‐, or 4‐week in vitro differentiation; note greatest mineralization density with longer differentiation period combined with CAM grafting. Exposure to vascularization at early time points is associated with accelerated mineralization compared with longer differentiation culture period in vitro. Scale bars = 50 μm, negative controls: 488 nm anti‐rabbit antibody only, **P < .01, ***P < .001, ****P < .0001. μCT, micro‐computed tomography; BV, bone volume; CAM, chorioallantoic membrane; COL1, Collagen‐1; hADSCs, human adipose‐derived stem cells; hBMSCs, human bone marrow‐derived mesenchymal stem cells; hOPs, human osteoprogenitor cells; TV, total volume

To study the effect of an in vivo environment on osteogenic differentiation of hADSCs, hOPs, and hBMSCs cultured in 3D fibrin, the cellularized scaffolds were CAM grafted for 7 days after culturing them for 3 weeks either in control or osteogenic differentiation media (n = 4 per condition, one cell line per lineage used; Figure 5A); comparison was made with undifferentiated and differentiated cellularized scaffolds maintained in culture for 3 weeks in vitro and not CAM‐grafted. At harvest, all CAM‐grafted constructs were encased by the CAM vasculature with evidence of neoangiogenesis into the engineered tissues (Figure S6).

Mineralization and osteoid formation were assessed by MacNeal's tetrachrome staining with Von Kossa counterstaining and the BV/TV calculated using ImageJ (Figure 5A). *In ovo* implantation of undifferentiated hOP and hBMSC scaffolds resulted in a significant increase in BV/TV (*P* = .029 and *P* = .075, respectively) when compared with control nongrafted scaffolds. Only nonsignificant changes in BV/TV were seen in hADSC and hBMSC scaffolds, between differentiated samples in vitro and their controls, or between CAM‐grafted differentiated samples and their controls. A significant increase was observed following CAM‐grafting osteogenic hADSC (*P* = .037) and hBMSC (*P* = .0054) compared with nongrafted undifferentiated counterparts Osteogenically differentiated hOPs had a significantly higher BV/TV compared with undifferentiated controls both prior to (*P* = .023) and following CAM‐grafting (*P* = .0016).

The extent of bone maturation in hADSCs induced to differentiate along the osteogenic lineage for different lengths of time in culture and subsequently vascularized was then assessed by COL1 immunofluorescent staining. hADSC fibrin scaffolds were maintained in culture in control or osteogenic differentiation media either for 2, 3, 4, or 5 weeks or for 2, 3, or 4 weeks and then CAM‐grafted for 7 days (Figure 5B‐D).

COL1 staining was more visible in hADSCs osteogenically differentiated in fibrin scaffolds in vitro when compared to their undifferentiated controls at all time points studied (Figure 5B). Pattern of expression changed from intracellular at 2 weeks of osteogenic differentiation to formation of an extensive fibrous network by 4 weeks.

hADSC‐fibrin scaffolds differentiated only in culture were then compared with scaffolds CAM‐grafted for 7 days following differentiation in culture for 2, 3, or 4 weeks (Figure 5C,D). The same endpoints were compared (eg, 4 weeks in vitro vs 3 weeks in vitro + 1 week in CAM) by μCT. 3D volume rendering of segmented μCT reconstructions of samples reveals calcium nodule formation in control and differentiated conditions in all groups before and after CAM‐grafting (Figure 5C). Surface models of calcification generated from the segmented images using the 3D viewer on ImageJ did not show increased mineralization with prolonged periods of differentiation in culture. In contrast, more surface calcification was observed in specimens CAM‐grafted after 3 weeks in differentiation medium than in those differentiated for 4 weeks in vitro. The extent of mineralization was assessed by measuring the BV/TV in μCT scans (Figure 5D). Cellularized scaffolds grafted onto the CAM showed increased BV/TV when osteogenically differentiated for 3 weeks (*P* < .0001) and 4 weeks (*P* < .0001), as compared to scaffolds differentiated in culture for the total corresponding time, 4 weeks and 5 weeks, respectively. A significant increase in BV/TV upon differentiation in vitro was observed only at 5 weeks. Furthermore, BV/TV was found to be higher at 4 and 5 weeks than at 3 weeks also in samples cultured in control media.

**FIGURE 6:**
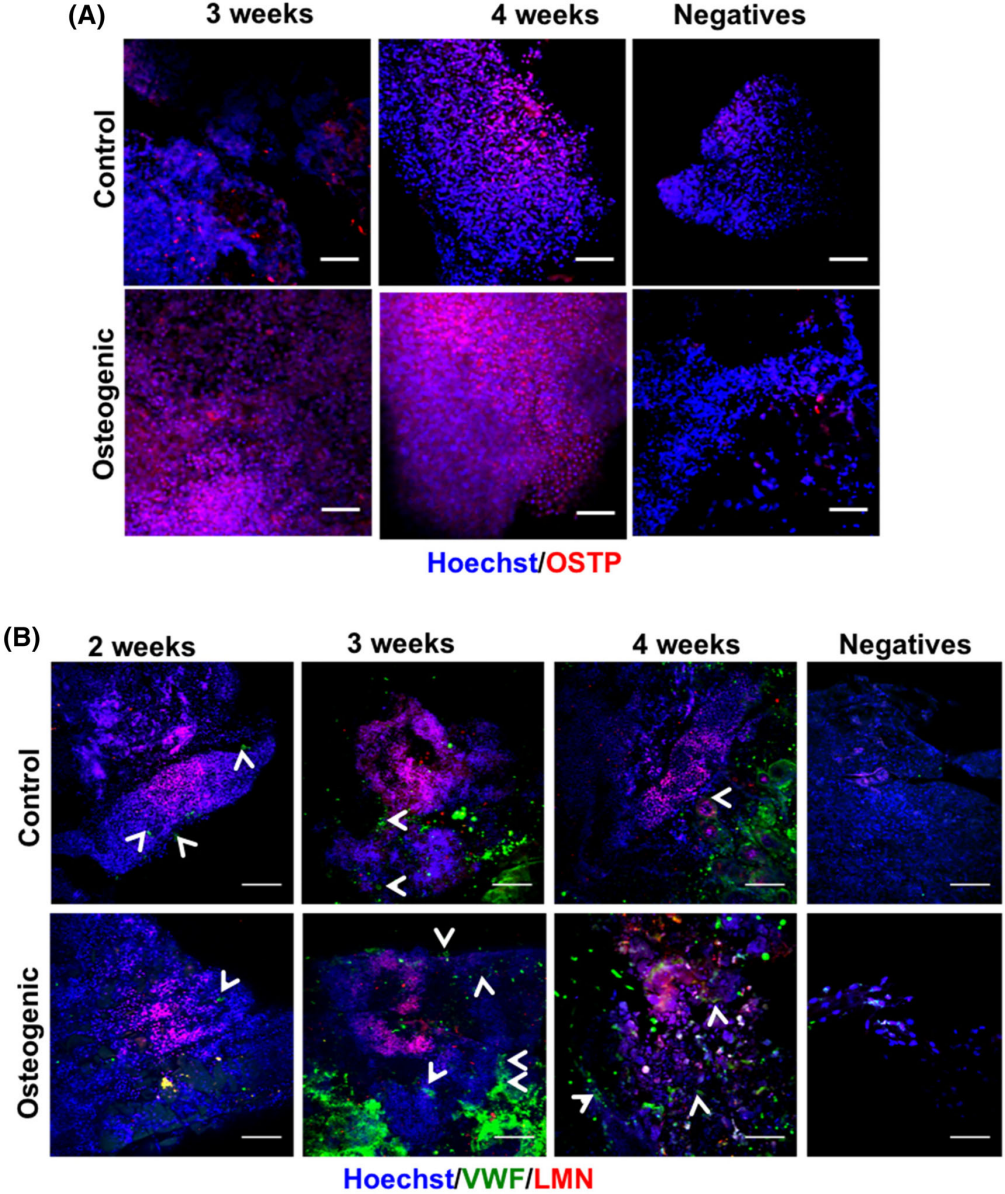
Detection of human cells, bone, and vascular markers by immunoflourescent in CAM‐grafted hADSC fibrin scaffolds following osteogenic induction in vitro. A, Immunoflourescent staining for osteopontin (OSTP) is more evident in osteogenically differentiated cells although longer period in control media prior to CAM‐grafting appears to induce more widespread OSTP expression. There is no visible difference in OSTP expression between 3 and 4 weeks of osteogenic differentiation. B, Immunoflourescent staining of whole mount samples for VWF at 2, 3, and 4 weeks of osteogenic differentiation reveals the presence of blood vessels (arrowheads point to lumens); human laminin A/C (LMN) indicates the presence of human cells within samples. Nuclei are detected by Hoechst dye staining. Scale bars = 100 μm, negatives: controls with both secondary antibodies only. CAM, chorioallantoic membrane. CAM, chorioallantoic membrane; hADSCs, human adipose‐derived stem cells; VWF, von Willebrand Factor

Immunostaining of hADSC‐fibrin scaffold CAM‐grafted after 3 and 4 weeks of osteogenic induction in vitro for osteopontin further supported osteogenic differentiation (Figure 6A). Extensive osteopontin staining was observed in the differentiated scaffolds, but some reactivity was also detected in samples maintained in control medium for 4 weeks prior to CAM‐grafting. Finally, to confirm the bone differentiation observed was due to the hADSCs present in the scaffolds and assess their vascularization, CAM‐grafted hADSC‐fibrin scaffold sections were labeled with a human‐specific antibody to lamin A/C and an antibody to von Willebrand factor (vWF) that reacts with both chick and human endothelial cells (Figure 6B). Cells positive for lamin A/C and cells only positive for vWF were detected within scaffolds maintained in control or osteogenic medium for 2, 3, or 4 weeks prior to grafting. This demonstrates survival of human cells in the scaffold over time as well as infiltration of host cells from the CAM consistent with neoangiogensis, as also shown by the stereoscopic imaging of *ex ovo* samples (Figure S6).

## 4 DISCUSSION

Here, we have defined biological properties of nonsyndromic pediatric calvarial bone for the first time. This has been used to compare osteogenic maturation of MSCs derived from this bone and hADSCs in 3D cultures. Furthermore, while previous work has shown that 3D cultures can accelerate osteogenic differentiation of human preosteoblasts,^30^ this is the first study demonstrating the positive effect of a 3D fibrin scaffold on osteogenic differentiation of pediatric MSCs from different sources over time. It also shows that short vascularization periods promote osteogenic maturation of these cells even without osteogenic stimulation in vitro prior to engraftment. Taken together, these data provide evidence that physiologically relevant conditions can drive hADSCs toward a more mature osteogenic phenotype, thus enhancing their potential for future therapeutic applications in bone regeneration.

### 4.1 Pediatric calvarial bone characterization establishes references of intramembranous bone biology and maturity

Calvarial bone grafts are commonly used as autologous implants for reconstruction of midfacial defects with an established safety and efficacy profile.^31^, ^32^ Thus, nonsyndromic pediatric calvarial bone represents a reasonable reference for evaluating bones bioengineered for pediatric craniofacial reconstruction. Previous bone characterization has most commonly looked at physical and biological properties separately and focused on samples from older patients.^33^, ^34^, ^35^ In defining the bone microenvironment and hierarchical structure at the macro and ultrastructural level, we have provided a clinically relevant standard against which bioengineered pediatric bone tissues can be evaluated.

However, the fact that for ethical reasons only bone from nonsyndromic craniosynostotic patient could be used, and that hOPs and hBMSCs were derived from this bone, has to be considered. While our results seem consistent with findings in normal adult bone,^36^ it is important to note that some differences in suture‐based gene expression (*FGF7*, *SFRP4*, and *VCAM1*) between nonsyndromic craniosynostotic patients, possibly related to vascularization, have been reported.^37^ Furthermore, higher alkaline phosphatase activity and reduced proliferation in sagittal craniosynostotic bone than in control was observed.^38^ Together, while the properties of craniosynostotic calvarial bone and the behavior hOPs and hBMSCs isolated from these samples appear to be similar to that of normal bones, they may not be entirely representative of bone and cells derived from healthy patients. Nevertheless, these cells could be suitable for tissue engineering, as an alternative to autologous bone grafts from these patients and would avoid additional surgery and morbidity. Equally worthy of consideration is that the tissues used to isolate hADSCs, while pediatric in nature, come from an older patient population (aged 7‐16 years) compared with the population the hOP and hBMSC were isolated from (3‐8 months). There was greater heterogeneity in the conditions presented by patients from whom hADSC lines were derived than in those of patients from whom bone tissue was harvested, all of whom had nonsyndromic sagittal craniosynostosis. While it has been recently shown that donor age does reduce hADSC proliferative and differentiation potential, at least into heapocytes, this study investigated only adult tissue donors (aged 22‐58 years).^39^ Pediatric hADSCs have been previously extensively studied by our group^12^, ^24^, ^40^, ^41^ and we have not observed any obvious difference in their behavior between donors of different ages, but no systematic analysis was carried out. To the best of our knowledge, there has been no published report on the impact of donor age on pediatric hADSC. While our hADSCs came from children with a more diverse set of clinical conditions, none of these conditions are known to affect bone formation or metabolism. It is important to note these differences and to appreciate in this context that the aim of this study was to evaluate hADSC suitability as sources for bone regeneration and demonstrate how this can be optimized.

### 4.2 hADSCs, hBMSCs, and hOPs have similar osteogenic potential but different capacity for mature bone formation

In addition to confirming previous studies indicating that hADSCs and hBMSCs are capable of self‐renewal and multilineage differentiation,^12^, ^24^, ^42^, ^43^ we show that also hOPs exhibit properties consistent with stemness, as indicated by expression of pluripotency markers, and tri‐lineage mesenchymal differentiation potential. hOPs express MSC markers in the same high percentage of cells as hADSCs and hBMSCs. Interestingly, hOPs also express nestin and vimentin. Beyond its original discovery as a neuroepithelial marker,^44^ nestin has been shown to be expressed also in hBMSCs^45^, ^46^ and hADSCs.^12^ Recently, nestin expression has been associated with a proangiogenic, multipotent population of BMSCs capable of self‐renewal and osteochondral differentiation in a mouse model.^47^ Vimentin is recognized as a MSC marker with proteomic analysis suggesting its association with migratory capability in porcine BMSCs.^48^ Relevant to the potential use of hOPs, hBMSCs, and hADSCs for allogeneic transplantation, HLA‐DR expression is very low in all three lines, and that of HLA‐ABC comparable. While it has been shown that HLA expression is similar in ADSCs and BMSCs,^49^ HLA expression in human osteoprogenitors derived from calvarial bone had not been previously reported. Given their relatively low immunogenicity, bioengineered bone from osteogenic MSCs, possibly HLA‐matched, might be suitable for allogeneic craniofacial reconstruction.

A key feature of osteoblast maturation is a change from an elongated to a cuboidal cell shape before acquiring the stellate appearance typical of osteocytes. Analysis of nuclear shape and size was one of the approaches used to monitor osteogenesis, as nuclear morphology is influenced by cellular geometry and changes in nuclear architecture and stiffness have been associated with osteogenic differentiation in animal and human MSCs, with the nuclear cytoskeleton believed to play a key role in these processes.^50^, ^51^, ^52^ Our combined approach, morphometric nuclear analysis, expression of COL1, and analysis of both organic and inorganic extracellular matrix, shows that all of the osteoprogenitor cells can undergo osteogenic differentiation in vitro, but the tissue generated by hBMSCs and hOPs presents more similarities to native bone concerning extracellular matrix deposition, consistent with differentiation observed in mouse osteoblasts.^53^, ^54^

### 4.3 3D culture and vascularization promote hADSC osteogenesis and accelerate bone maturation

Engineering autologous bone using hADSCs is an attractive prospect for pediatric craniofacial reconstruction as they are an easily accessible and abundant stem cell source. Their use, however, has been limited by the fact that hADSC‐derived bone both in vitro and in vivo tends to be less mature when compared to bone derived from osteoprogenitor cells. Here, we have shown that culturing hADSCs, as well as hBMSCs and hOPs, in a 3D fibrin scaffold augments bone differentiation and stimulates extracellular deposition of COL1 even in undifferentiated cultures. Increased COL1 is consistent with an increase in nodule formation in osteogenically induced hADSCs, presumably as a consequence of increased condensation, a crucial step in osteogenesis that is required for osteoprogenitor proliferation and their subsequent differentiation.^55^ Furthermore, this study shows that some significant differences in Collagen 1 gene expression observed among the cell lines in both control and osteogenic conditions disappear when the cells are cultured in 3D. Indeed, all cell lines studied here showed enhanced osteogenic differentiation in 3D fibrin cultures, though with some differences. While the orientation and alignment of collagen networks in differentiated hADSCs and hOPs is similar, as indicated by their coherency coefficients, the matrix deposited by hBMSCs appears to achieve a higher level of organization. In contrast, the BV/TV is very similar in differentiated hADSCs and hBMSCs, but lower than in hOPs. This is in line with a previous report suggesting that human preosteoblast differentiation and maturation is accelerated in human preosteoblast cells cultured as micromasses.^30^ Together, culturing hADSCs in fibrin scaffolds enhances their osteogenic differentiation, making them more similar to the other lines we have investigated. Nonetheless, none of the osteogenic cultures currently produced by any of these cells in vitro achieves the level of organization of native calvarial bone.

A further step toward increasing hADSC‐derived bone maturation is vascularization, and for the first time we have assessed the effect of vascularization at different times after in vitro osteogenic induction. Blood supply is vital to bone formation and previous work has suggested that human preosteoblast differentiation is most vulnerable to oxygen deprivation at the stage of matrix maturation.^56^ Indeed, our CAM grafting studies show that early vascularization of hADSCs enhances bone maturation even more than prolonged in vitro culture in osteoinductive media. This extends previous studies where mineralization and mature bone formation was found to be increased by mimicking vascularization in vitro in cocultures of endothelial cells and human MSC.^57^, ^58^, ^59^, ^60^ It is also important to consider that ADSCs have previously been shown to exhibit a proangiogenic effect via release of angiogenic factors such as vascular endothelial growth factor (VEGF), differentiate into endothelial cells in vitro and improve postnatal neovascularization in vivo.^61^, ^62^, ^63^ The data shown here further support the proangiogenic potential of these cells which then augments their osteogenic maturation capabilities.

Together, our results show that 3D culture and vascularization play a key role in hADSC‐derived osteogenic differentiation and maturation. This effect is enhanced by preincubation in osteoinductive media and altogether hint toward adopting a more global approach combining chemical, physical, and biological stimulation for bone tissue engineering.

## 5 CONCLUSIONS

While further work is required to achieve the complex architecture of native calvarial bone, we have demonstrated that differentiation of hADSCs can be enhanced by changing the physical environment through a simple 3D culture system and early vascularization. The ability to drive hADSC osteogenic maturation has profound implications for enabling their clinical use for autologous reconstruction of pediatric bone. Development of more complex scaffolds to help recreate the 3D bone microenvironment and longer vascularization periods in vivo will be crucial to achieving this goal.

## CONFLICT OF INTEREST

The authors declared no potential conflicts of interest.

## AUTHOR CONTRIBUTIONS

A.I.: experiment design, performed experiments, data analysis, manuscript writing, obtaining funding; N.R.‐F.: μCT analysis of the bone samples, obtaining patient consent, collection of tissues, critical reading of the manuscript; E.Z., O.F.W.G., S.E.P.N.: generation of cell lines, critical reading of the manuscript; A.B., D.D., N.W.B.: obtaining patient consent, collection of tissues, critical reading of the manuscript; P.F.: research supervision, data analysis, obtaining funding, manuscript writing.

## Supporting information


**Appendix** S1: Supporting information

## Data Availability

The data that support the findings of this study are available from the corresponding author upon reasonable request.
