# Supplementary material for: Three‐dimensional environment and vascularization induce osteogenic maturation of human adipose‐derived stem cells comparable to that of bone‐derived progenitors
Source: Stem Cells Transl Med. 2020 Jul 8;9(12):1651–66. doi: 10.1002/sctm.19-0207 (PMC7695642; doi:10.1002/sctm.19-0207)
Supplement: Supplementary file 1 — Appendix S1: Supporting information [file SCT3-9-1651-s001.docx]

## SUPPLEMENTARY DATA

Supplementary Table 1: Details of cell lines used.

| Cell line | Cell type | Age | Gender | Patient diagnosis |
| --- | --- | --- | --- | --- |
| H11 | hADSC | 16 years | M | Craniofacial microsomia |
| H12 | hADSC | 16 years | M | Gynecomastia |
| H13 | hADSC | 17 years | F | Goldenhar syndrome |
| H20 | hADSC | 12 years | F | Linear Morphea/Parry Romberg syndrome |
| H24 | hADSC | 9 years | F | Craniofacial microsomia |
| H33 | hADSC | 15 years | F | Lipoma |
| H41 | hAEDSC | 9 years | F | Goldenhar syndrome |
| H44 | hOP/hBMSC | 5 months | M | NSSC |
| H45 | hOP/hBMSC | 8 months | M | NSSC |
| H46 | hAEDSC | 16 years | F | Cavernous haemangioma |
| H47 | hAEDSC | 9 years | M | Parry Romberg syndrome |
| H49 | hOP/hBMSC | 6 months | M | NSSC |
| H52 | hOP | 6 months | M | NSSC |
| H55 | hOP/hBMSC | 5 months | M | NSSC |
| H64 | hOP | 6 months | F | NSSC |
| H67 | hOP | 8 months | F | NSSC |
| H69 | hAEDSC | 15 years | M | Goldenhar syndrome |
| H70 | hOP/hBMSC | 3 months | M | NSSC |
| H71 | hOP/hBMSC | 7 months | M | NSSC |
| H72 | hOP/hBMSC | 7 months | F | NSSC |
| H75 | hOP | 3 months | M | NSSC |
| H87 | hAEDSC | 16 years | M | Parry Romberg |
| H88 | hAEDSC | 7 years | F | Parry Romberg |

hAEDSC; human adipose explant dedifferentiated stem cells, hADSC; human adipose derived stem cells, hOP; human osteoblast precursors, hBMSC; human bone marrow derived stem cells; M, male; F; female; NSSC; Non syndromic sagittal craniosynostosis.

Supplementary Table 2: Details of primers used for PCR.

| Gene | Forward (FW) and Reverse (REV) Primers (5’-3’) | Anneal  temp (°C) | Cycles | Product size (bp) |
| --- | --- | --- | --- | --- |
| CMYC | FW CTTCCCCTACCCTCTCAACG  REV AGTGGGCTGTGAGGAGGTTT | 56 | 35 | 303 |
| COL1 | FW ATGCCTGGTGAACGTGGT  REV AGGAGAGCCATCAGCACCT | 56 | 26 | 87, 240, 267 |
| KLF-4 | FW CCCACACAGGTGAGAAACCT  REV TTCTGGCAGTGTGGGTCATA | 56 | 35 | 220 |
| L19 | FW GCGGAAGGGTACAGCCAAT  REV CAGGCTGTGATACATGTGGCG | 56 | 32 | 130 |
| NES | FW CAGCGTTGGAACAGAGGTTGG  REV TGGCACAGGTGTCTCAAGGGTAG | 56 | 35 | 389 |
| NANOG | FW GGATGGTCTCGATCTCCTGA  REV CCTCCCAATCCCAAACAATA | 56 | 35 | 252 |
| SOX2 | FW CATGTCCCAGCACTACCAGA  REV GTCATTTGCTGTGGGTGATG | 56 | 35 | 234 |
| VIM | FW GACACTATTGGCCGCCTGCAGGATGAG  REV CTGCAGAAAGGCACTTGAAAGC | 56 | 35 | 418 |

Supplementary Table 3: Antibodies used in flow cytometry.

| **Antibody** | **Antigen** | **Fluorophore** | **Dilution** |
| --- | --- | --- | --- |
| CD10 | neprilysin | FITC | 1 in 5 |
| CD13 | aminopeptidase N3 | PE | 1 in 5 |
| CD14 | monocyte differentiation antigen | FITC | 1 in 5 |
| CD29 | integrin B1 | PE | 1 in 5 |
| CD31 | platelet endothelial cell adhesion molecule-1 | FITC | 1 in 5 |
| CD34 | hematopoietic progenitor cell antigen | FITC | 1 in 50 |
| CD44 | hyaluronic acid receptor | PE | 1 in 5 |
| CD45 | lymphocyte common antigen | FITC | 1 in 10 |
| CD73 | ecto-5’-nucleotidase | PE | 1 in 5 |
| CD90 | Thy1 |  |  |
| CD105 | endoglin | PE | 1 in 20 |
| CD117 | c-kit | PE | 1 in 5 |
| CD166 | activated leucocyte cell adhesion molecule | PE | 1 in 5 |
| HLA ABC | Human leukocyte antigen ABC | PE | 1 in 5 |
| HLA DR | Human leukocyte antigen DR | FITC | 1 in 5 |
| IgG1 | Immunoglobulin G1 | FITC | 1 in 5 |
| IgG1 | Immunoglobulin G2 | PE | 1 in 5 |
| IgG2a | Immunoglobulin G2a | FITC | 1 in 5 |
| IgG2b | Immunoglobulin G2b | FITC | 1 in 5 |

All antibodies were purchased from BD biosciences.

Supplementary Table 4: Antibodies and dyes used for protein detection and nuclear staining in immunocytochemistry experiments.

| **Primary Antibodies – target proteins** | Species | Dilution | Source |
| --- | --- | --- | --- |
| Vimentin | Mouse | 1:100 | DAKO |
| Osteopontin | Mouse | 1:200 | Hybridoma bank |
| Lamin A/C | Mouse | 1:100 | Abcam |
| Nestin | Rabbit | 1:100 | Millipore |
| Collagen 1 | Rabbit | 1:100 | NOVUS |
| Von Willebrand Factor | Rabbit | 1:5000 | DAKO |
| **Secondary Antibodies** |  |  |  |
| Anti-Mouse IgG Alexa Fluor 568/594 | Goat | 1:400 | Invitrogen |
| Anti-Rabbit IgG Alexa Fluor 488 | Goat | 1:400 | Invitrogen |
| **Dyes** |  |  |  |
| Fluorescein Phalloidin (F-Actin) | - | 1:400 | Thermo Fisher Scientific |
| Hoechst 33258 (Nuclear staining) | - | 1:400 | Thermo Fisher Scientific |


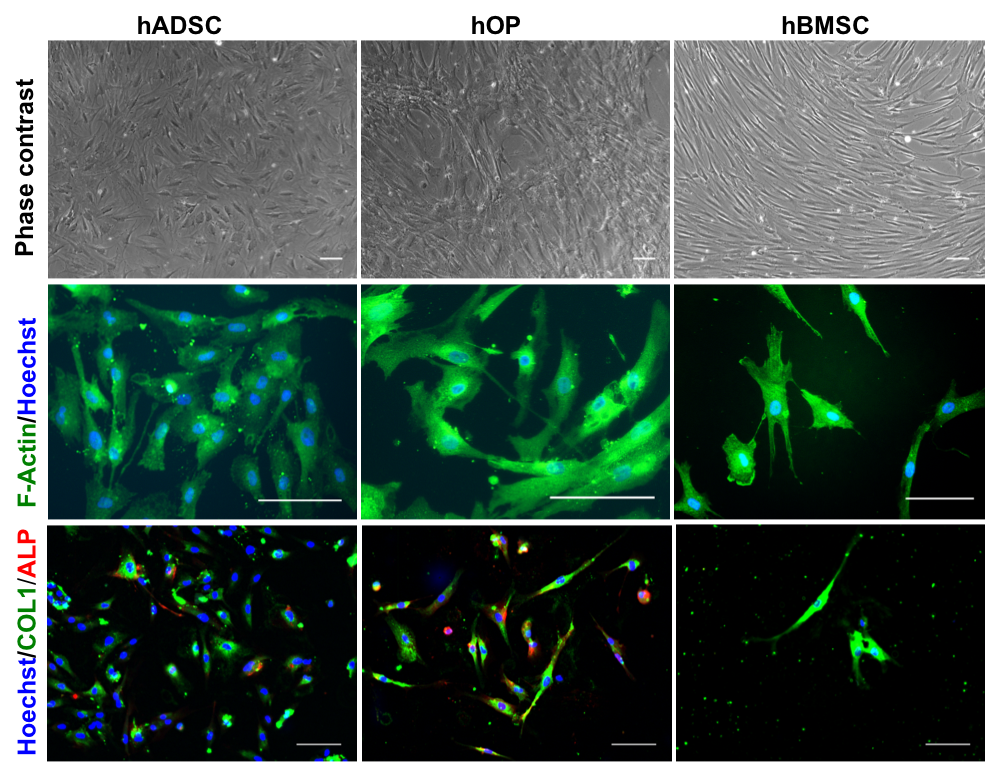


Supplementary Fig 1: Comparison of different osteoprogenitor cell morphology by phase contrast imaging and immunocytochemistry.

Typical appearance of confluent monolayer cultures on plastic of hADSCs (n=6), hOPs (n=11) and hBMSCs (n=7) through phase contrast microscopy with F-actin (green) and Hoechst nuclear dye (blue) staining of cells scale bars: 100 µm.


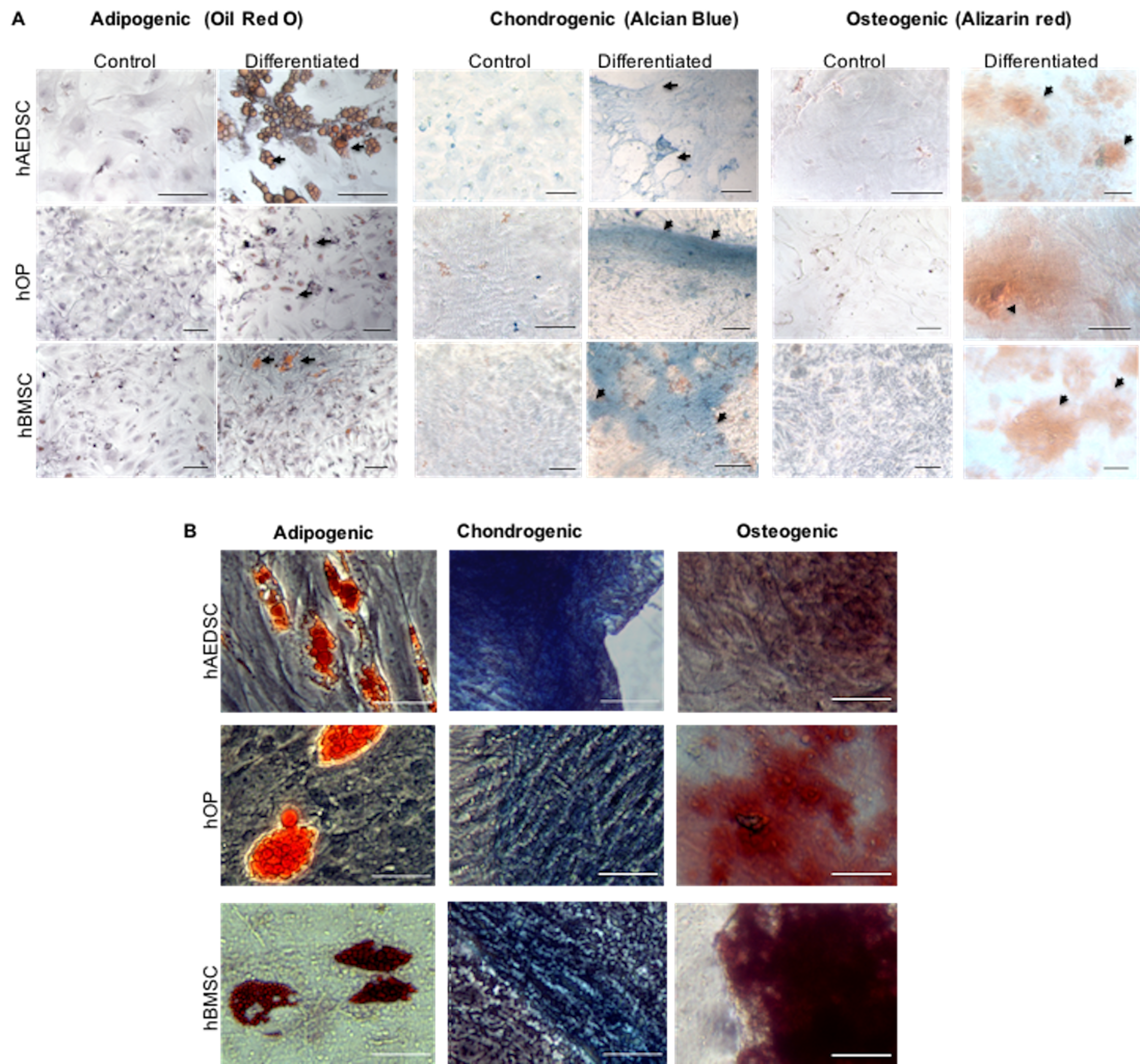


Supplementary Fig 2: Trilineage differentiation of hADSCs, hOPs and hBMSCs in monolayer cultures.

A) Adipogenic differentiation is detected using Oil Red O and nuclei are counterstained with haematoxylin; arrows indicate oil droplets. Alcian blue staining is used to detect chondroid matrix deposition (arrows). Osteogenic differentiation is assessed by Alizarin red staining; arrows indicate mineral nodule formation. Note that alcian blue staining appears to be more intense in hOPs and hBMSCs than hADSCs, while more lipid droplets are present in adipogenically differentiated hADSCs than in the other lines; mineralization following osteogenic induction seems more extensive in hOPs. B) Higher magnification of differentiated cells undergoing Oil Red O for adipogenic, Alcian blue for chondrogenic and Alizarin red for osteogenic differentiation. Scale bars: A; 100 µm, B; 50 µm.


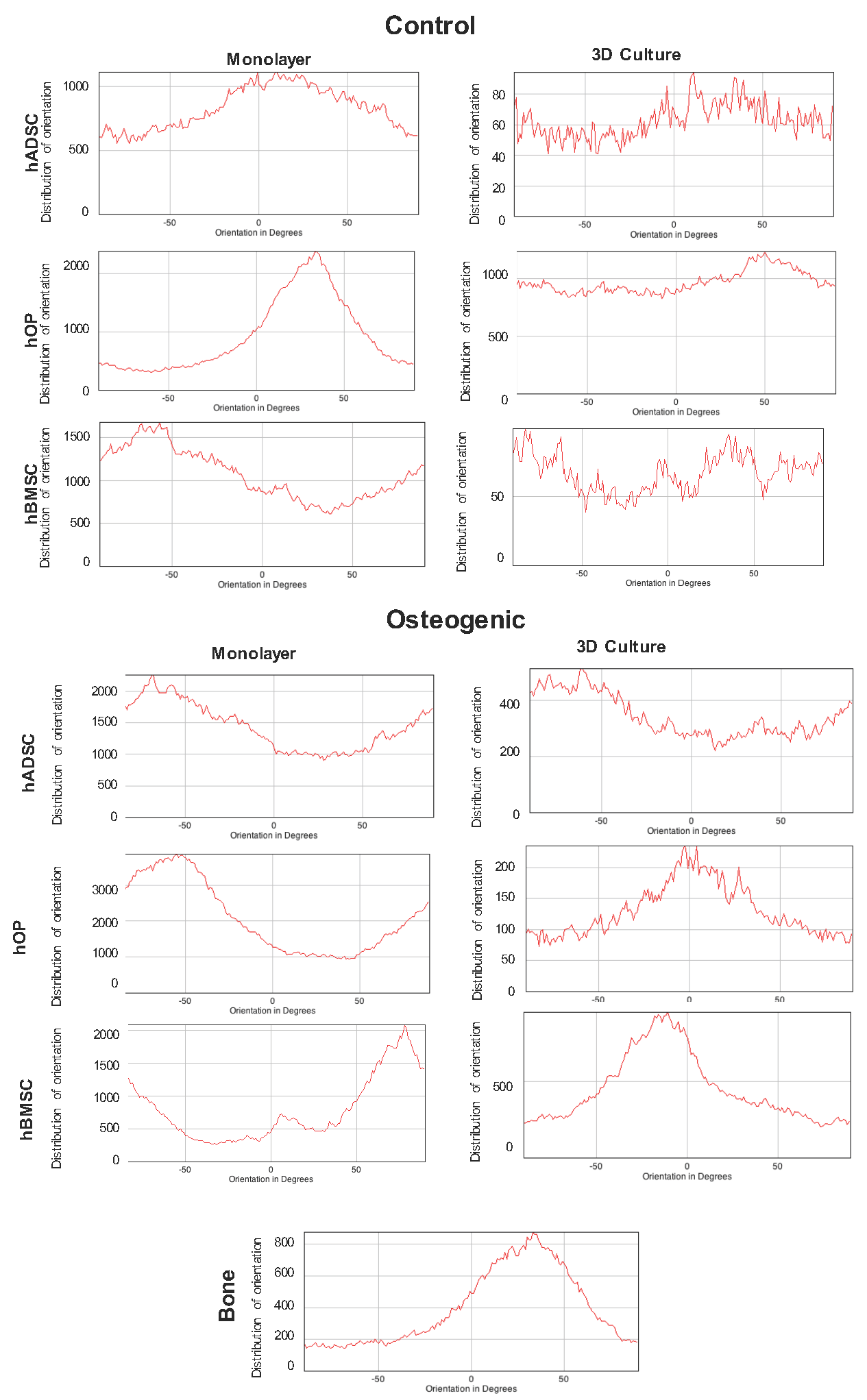


Supplementary Fig 3: Comparison of the impact of osteogenic differentiation on production of organized extracellular collagen fibers in 3D cultures of hADSCs, hOPs and hBMSC.

Histograms plot the distribution of the orientations of collagen fibres deposited by paediatric stem cells with osteogenic potential. Calvarial bone provides a positive control for mature collagen fibre orientation where a distinct uniform peak is seen. Disordered fibrillar patterning is suggested by irregular and indistinct peaks which suggests multiple and varying fibre distributions. This pattern is observed in both undifferentiated and differentiated 3D hADSC fibres which show disordered orientations as evidenced by wide distributions on the histogram. In contrast, differentiated hOPs and hBMSCs show an isotropic fibre orientation (see sharp narrow histogram peak) suggesting expression of organised extracellular collagen.

*
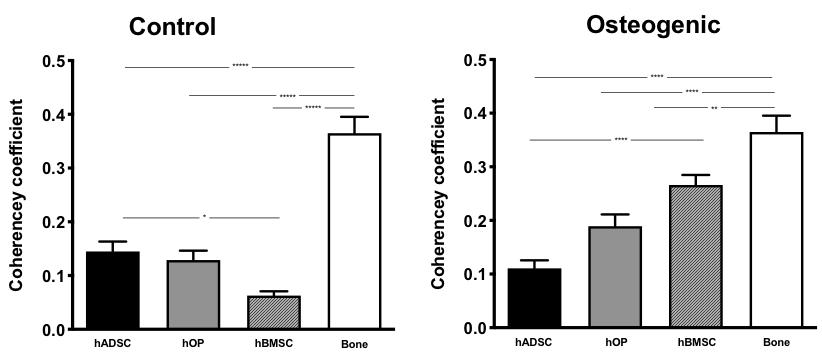
*

Supplementary Fig 4: Comparison of collagen fibre coherency between control and differentiated hADSC, hOP and hBMSC cultured on 3D fibrin gels for 3 weeks.

3D Coherency coefficient isolated from OrientationJ using uniform weights gives a measure of collagen directionality in hADSCs, hOPs and hBMSCs as compared to bone positive control. Scale bar; 100 µm, data represented as mean ± SEM, p-values; *p<0.05; **p<0.01; ***p<0.001; ****p<0.0001.


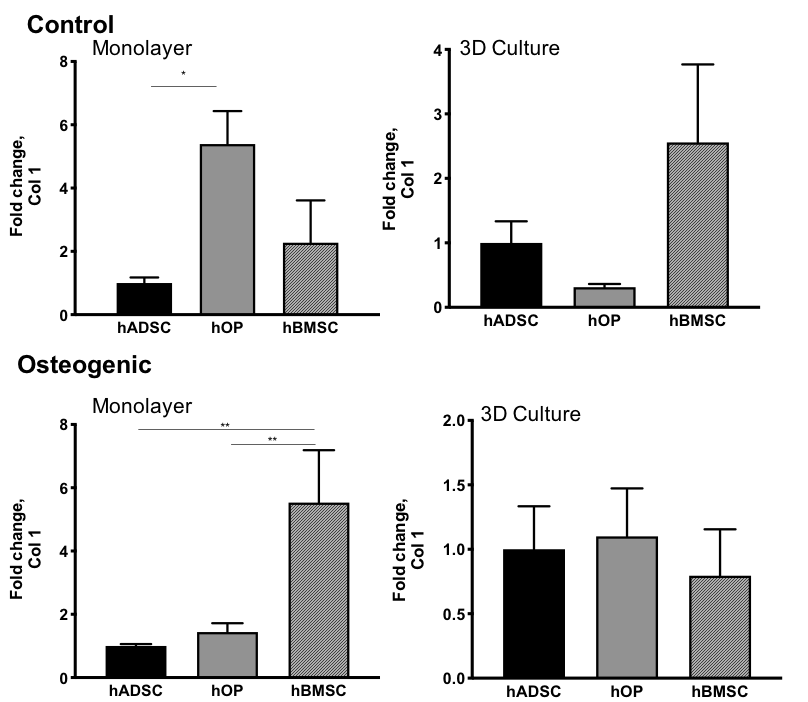


Supplementary Fig 5: Collagen 1 gene expression by RT-qPCR in hADSCs, hOPs and hBMSCs.

Gene expression represented as fold change relative to hADSC. Scale bar; 100 µm, data represented as mean ± SEM, p-values; *p<0.05; **p<0.01.


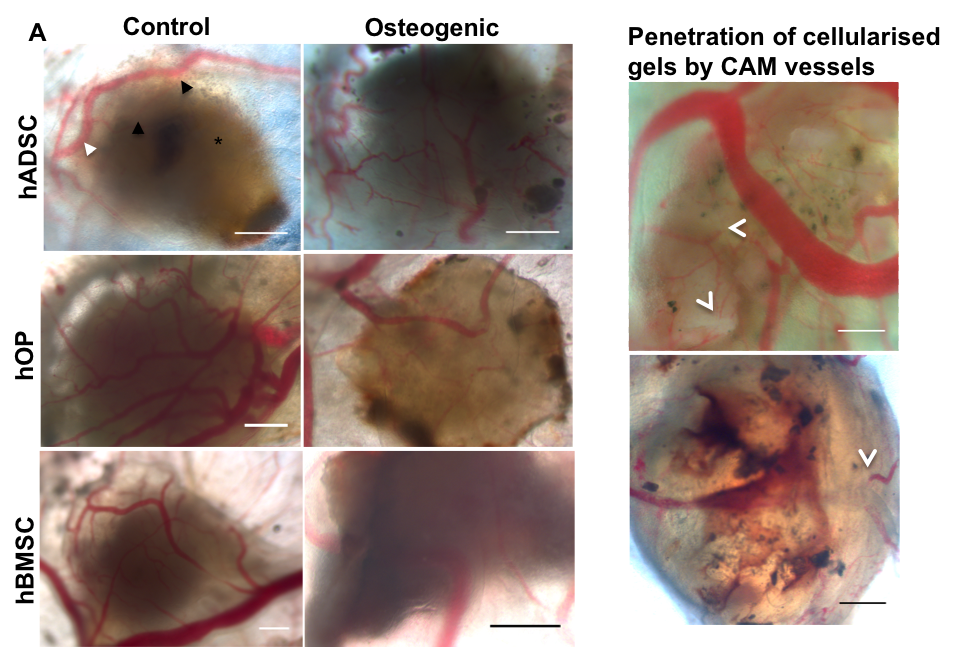


Supplementary Fig 6: Vascularization of hADSC, hOP and hBMSC fibrin scaffolds following chorioallantoic membrane (CAM) grafting.

In ovo images taken on the day of harvest showing the occurrence of vascularisation (*) in hADSC, hOP and hBMSC fibrin scaffolds cultured in control or osteogenic media for 3 weeks and then CAM-grafted for 7 days; the white arrowhead indicates blood vessel; black arrowheads indicate microvessel formation. On the right-hand side are higher magnifications demonstrating vessels penetrating the cellularised scaffolds from the CAM (white arrows). Scale bars: 0.5mm.
